# Changes of intestinal microbiota in the giant salamander (*Andrias davidianus*) during growth based on high-throughput sequencing

**DOI:** 10.3389/fmicb.2023.1052824

**Published:** 2023-03-16

**Authors:** Mingcheng Cai, Huan Deng, Hanchang Sun, Wantong Si, Xiaoying Li, Jing Hu, Mengjun Huang, Wenqiao Fan

**Affiliations:** ^1^Institute of Aquatic Animal Disease Prevention and Control, Chongqing University of Arts and Sciences, Chongqing, China; ^2^Chongqing Key Laboratory of Kinase Modulators as Innovative Medicine, Chongqing, China; ^3^Chongqing Engineering Laboratory of Targeted and Innovative Therapeutics, Chongqing, China

**Keywords:** giant salamander, growth, intestinal microflora, 16S rRNA sequencing, health indicator

## Abstract

Despite an increasing appreciation of the importance of host–microbe interaction in healthy growth, information on gut microbiota changes of the Chinese giant salamander (*Andrias davidianus*) during growth is still lacking. Moreover, it is interesting to identify gut microbial structure for further monitoring *A. davidianus* health. This study explored the composition and functional characteristics of gut bacteria in different growth periods, including tadpole stage (ADT), gills internalization stage (ADG), 1 year age (ADY), 2 year age (ADE), and 3 year age (ADS), using high-throughput sequencing. The results showed that significant differences were observed in microbial community composition and abundance among different growth groups. The diversity and abundance of intestinal flora gradually reduced from larvae to adult stages. Overall, the gut microbial communities were mainly composed of *Fusobacteriota*, *Firmicutes*, *Bacteroidota*, and *Proteobacteria*. More specifically, the *Cetobacterium* genus was the most dominant, followed by *Lactobacillus* and *Candidatus Amphibiichlamydia.* Interestingly, *Candidatus Amphibiichlamydia*, a special species related to amphibian diseases, could be a promising indicator for healthy monitoring during *A. davidianus* growth. These results could be an important reference for future research on the relationship between the host and microbiota and also provide basic data for the artificial feeding of *A. davidianus*.

## 1. Introduction

The Chinese giant salamander (*Andrias davidianus*), widely distributed in China, is the largest and most primitive urodele amphibian alive worldwide. It has been listed as a national class II protected species in China and the Appendix I of the Convention on International Trade in Endangered Species of Wild Fauna and Flora (CITES), 2008 (Zhang et al., 2003; Jiang et al., 2015; Turvey et al., 2021). With the breakthrough of artificial breeding technology, the *A. davidianus* aquaculture industry is rapidly developing. Giant salamander aquaculture could not only meet the consumption needs of modern life but also promote economic revitalization in remote mountains, which is listed as a key project for agricultural industrialization. In China, there were ~2 million *A. davidianus* artificially reproduced each year (Meng et al., 2014). The breeding modes of this species mainly included entire captive, bionic captive, and primordial ecological breeding (Liang, 2007). Under artificial breeding conditions, *A. davidianus* generally could grow to the adult stage in 2–3 years. However, the morbidity of infectious diseases is increasing due to intensive culture and artificial changes in the living environment. Infectious diseases mainly included bacterial, viral, parasitic, and other diseases, which could cause heavy economic losses (Jiang et al., 2015; Gui et al., 2018). At present, it is difficult to evaluate whether *A. davidianus* grows healthily and to choose reasonable measures to prevent relative diseases at the early stage. Hence, it is important to understand the health status and select an accurate monitoring index to guide the breeding of *A. davidianus*.

The gut microbiota plays important roles in host metabolism and immunity, making it an effective indicator for monitoring the response of animal organisms to environmental changes (Tilg and Kaser, 2011). The gut microbes are diverse, mainly live in the second half of the digestive tract, and consist of nearly 200 common species and about 1,000 uncommon species (Ley et al., 2008). Factors such as host diets, genetic background, and immune status could influence microbiota composition (Turnbaugh et al., 2009; Benson et al., 2010). The population and abundance of gut microbes in different habitats vary greatly, and the status also is reflected in the giant salamander under different temperatures and ages (Zhang et al., 2018; Zhu et al., 2021). There is a close relationship between intestinal flora and host health (Wu et al., 2019), which mainly reflected that microbes are mainly colonized in the intestinal tract and play key roles in the digestive system (Gerritsen et al., 2011). The complex microbes constitute a microbial community, and the balance contributes to maintaining the host’s gut function, including energy uptake, metabolite production, immune system development, and gastrointestinal diseases (He, 2012).

According to statistics, <1% of microorganisms in nature could be obtained by the traditional media method *in vitro.* High-throughput sequencing technology is widely used to detect the gut microecosystem considering the complex and culture-independent feature (Turnbaugh et al., 2010; Siezen and Kleerebezem, 2011). However, there are insufficient profiles and changes of gut microbiota in the Chinese giant salamander, which results in a lack of comprehensive understanding of the relationship between the microbiome and host considering diverse species.

This study mainly focused on the monitoring indices of *A. davidianus* during the breeding process. Intestinal microbes of healthy *A. davidianu*s in different growth periods were collected for 16S rRNA sequencing to understand the changes in intestinal microflora from larvae to adult stages. The results contributed to establishing a gut microbiota database for *A. davidianus* and provided much information for the construction of an accurate monitoring system and standardized artificial breeding of *A. davidianus*.

## 2. Materials and methods

### 2.1. Animals breeding and sample collection

A total of 80 healthy *A. davidianus* were cultured in the Experimental Animal Center at Chongqing University of Arts and Sciences. The growth data of *A. davidianus*, such as body length and weight, were recorded during whole growth periods. According to the growth characteristics, the guts of *A. davidianus* were collected from five growth stages, which include tadpole stage (ADT), gills internalization stage (ADG), 1 year age (ADY), 2 year age (ADE), and 3 year age (ADS). Three *A. davidianus* were randomly selected for euthanasia in each group, and the gut was excised from the abdominal cavity. The separated guts were transferred to a sterilized kraft paper and knotted with cotton rope to decrease the cross-pollution in the different intestinal segments. The tissue samples were then immersed in a 4% paraformaldehyde solution for fixation. The contents at the end of the rectum were immediately collected and stored at −80°C until further high-throughput sequencing analysis.

### 2.2. Histomorphological observation of guts

The tissue samples of the small intestine, large intestine, and rectum collected at different growth stages were sectionized using the conventional paraffin ultra-thin sectioning method (Schmitt et al., 2019). This process mainly includes embedding, sectioning, and HE staining steps. After completion, the morphological changes of intestinal tissue at each stage were observed with a microscope and photographed using a microscopic imaging system.

### 2.3. DNA extraction

To analyze the composition of the bacterial community, genomic DNA was extracted from the aforementioned gut content samples using the CTAB method (Wilson, 2001). DNA extraction operation was quickly performed after these gut samples were fully mixed. Agarose gel (0.8% w/v) electrophoresis was then performed to evaluate the purity and concentration of the extracted DNA. According to the concentration detected, an appropriate amount of DNA was taken and diluted to 1 ng/μL with sterile water.

### 2.4. 16S rRNA amplification and sequencing

Using diluted genomic DNA as a template, specific primers with barcodes were designed according to the selection of sequencing regions. The 16S rDNA target region (V3/V4) was amplified by PCR with primers 338F: 5′-ACTCCTACGGGAGGCAGCA and 806R: GGACTACHV GGGTWTCTAAT-3′. More detailed parameters of the PCR reaction were performed as described previously (Song et al., 2019). PCR products were detected by electrophoresis with agarose gel (2% w/v). The PCR products were purified by magnetic beads, quantified by a microplate reader, and then mixed in equal amounts according to concentration. After full mixing, PCR products were detected by electrophoresis with agarose gel (2% w/v). DNA fragments from the agarose gels were recovered using a QIAquick Gel Extraction Kit. The purified PCR products were used for constructing the sequencing library using TruSeq^®^ DNA PCR-Free Sample Preparation Kit. Prior to sequencing, the sequencing libraries were subjected to quantification using Qubit and Q-PCR. The qualified libraries were subjected to high-throughput sequencing using an Illumina NovaSeq 6000.

### 2.5. Bioinformatics and statistical analysis

The paired-end sequences from high-throughput sequencing were assigned to the corresponding samples according to the primer and barcode information. After amputation of barcode and primer sequences, the reads of each sample were spliced to obtain raw tags using FLASH (version 1.2.7; Magoč and Salzberg, 2011) and were then strictly filtrated to obtain clean tags (Bokulich et al., 2013). The quality of raw tags was evaluated using Quantitative Insights into Microbial Ecology (QIIME) software (version 1.9.1; Caporaso et al., 2010). For raw tag interception, the threshold value of continuous low-quality bases is 19, and the base length is 3. These tags would be filtered when the base length is <75% of whole tags with continuous high quality. Finally, the effective tags were obtained when raw tags, such as ambiguous bases, chimeras, and mismatched primers in the reads, were filtered by initial quality screening. The obtained high-quality effective tags for all samples were clustered in operational taxonomic units (OTUs) based on 97% identity using the UPARSE algorithm (version 7.0.1001.). Prior to homogenization, the representative sequences for OTUs were annotated and classified based on the Mothur method and SSU rRNA database and were phylogenetically analyzed by MUSCLE (version 3.8.31; Quast et al., 2013). Alpha diversity analysis was performed using QIIME (version 1.9.1) to calculate the values of observed-OTUs, Chao1, Shannon, Simpson, and so on. Beta diversity was used to identify similarities and differences between different samples using the same QIIME software. In addition, rarefaction curves were used to evaluate the rationality of sequencing depth. Linear discriminant analysis effect size (LEfSe) was generated to identify significantly differential biomarkers among groups. Based on the microbiota composition, the functional enrichment of the KEGG pathway was further predicted by PICRUSt2 (version 2.3.0-b; Langille et al., 2013). R software (version 2.15.3) was applied to statistical analysis and plotting. The criterion of significance was conducted at *p*-values of <0.05, and the data were expressed as means ± SD.

## 3. Results

### 3.1. Growth and histomorphology changes in *Andrias davidianus*

Five sampling time points were chosen, namely, tadpole stage (ADT, 90 days), gills internalization stage (ADG, 180 days), 1 year age (ADY, 365 days), 2 year age (ADE, 730 days), and 3 year age (ADS, 1,095 days). We measured the body weights and lengths of the Chinese giant salamander and collected their gut samples during growth. Average weights of *A. davidianus* were 5.03 ± 0.21, 65.17 ± 11.28, 175.50 ± 29.86, 1640.07 ± 86.51, and 2450.93 ± 125.61 g for ADT, ADG, ADY, ADE, and ADS, respectively. The total lengths of 80 individuals were 4.13 ± 0.31, 29.10 ± 1.15, 40.87 ± 3.59, 67.63 ± 2.90, and 75.37 ± 3.37 cm for the aforementioned stages.

**Figure 1:**
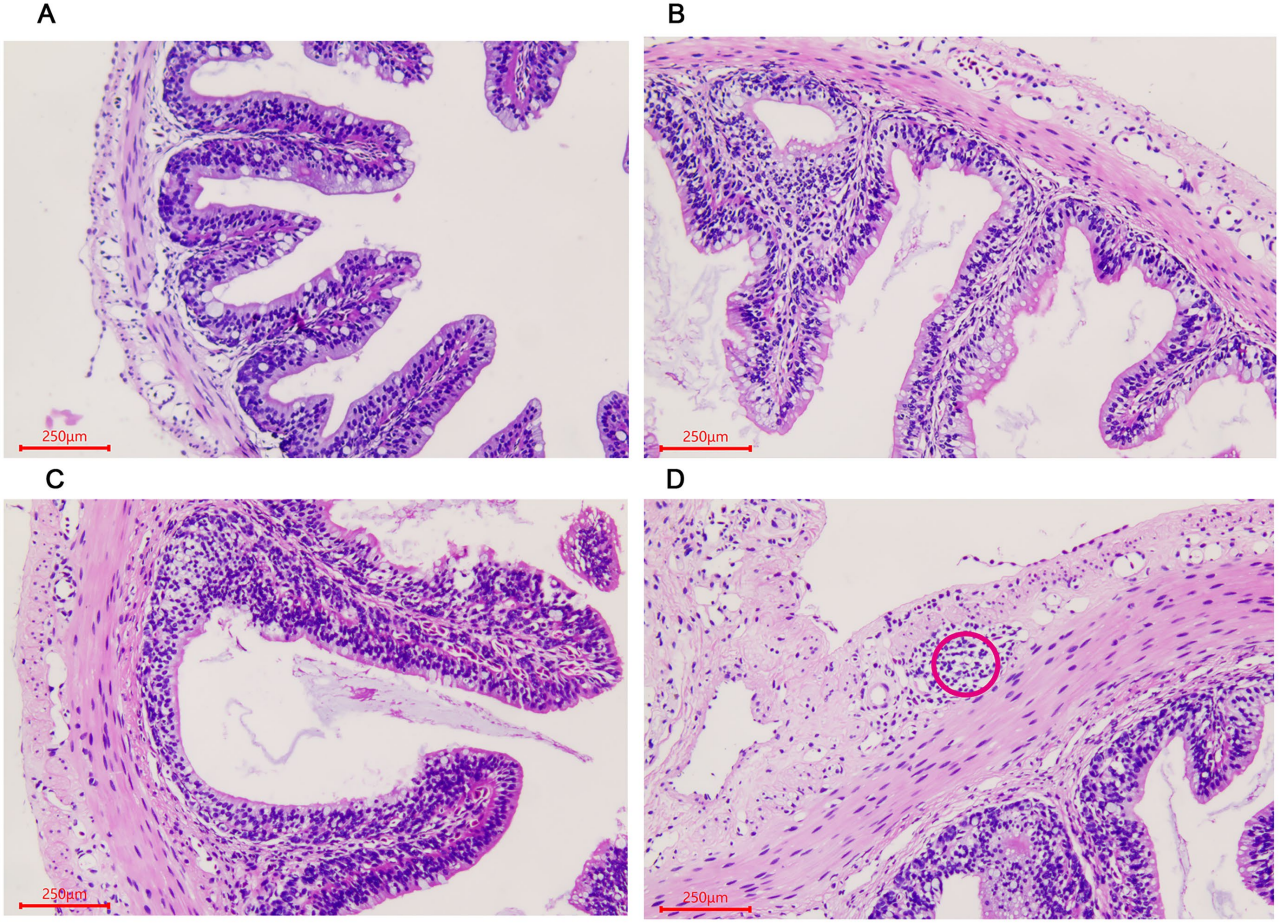
Morphological changes of the small intestine in *Andrias davidianus.* **(A)** The small intestine in ADY. **(B)** The small intestine in ADE. **(C)** The small intestine in ADS. **(D)** Lymphoid follicular cluster of the small intestine in ADS (as shown in red circle). The sections were observed using high power fields of 500-fold magnification with several measurements at different positions in each sample.

The histological changes of the *A. davidianus* gut at different growth periods were observed using a microscope after staining the tissue section with hematoxylin–eosin (HE). In the small intestine, both muscular thickness and villi length gradually increased with the increase in age, as shown in ADY, ADE, and ADS stages (Figures 1A–C). Lymphoid follicular accumulation began to appear in the submucosa in the ADS stage (Figure 1D).

**Figure 2:**
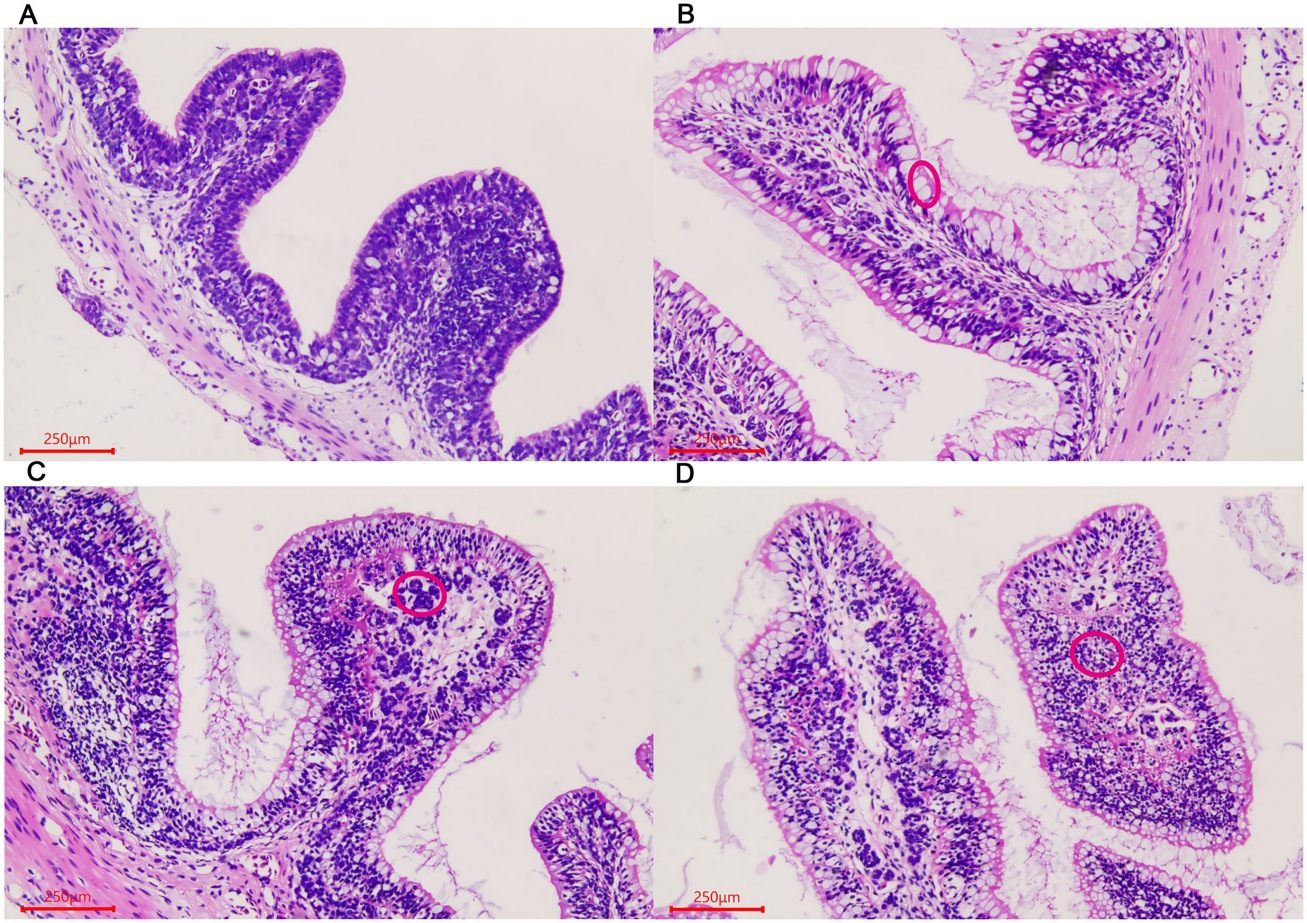
Morphological changes of the large intestine in *A. davidianus.* **(A)** The large intestine in ADY. **(B)** The large intestine in ADE. **(C)** The large intestine in ADS. **(D)** Glandular structures of the large intestine in ADS (as shown in red circle). The sections were observed using high power fields of 500-fold magnification with several measurements at different positions in each sample.

Similar changes were observed in the large intestine in the three stages. The number of goblet cells gradually increased in the villous epithelium with the growth of the giant salamander (Figures 2A–C). Gland-like structures were seen in the lamina propria of villi in the ADS sample (Figure 2D). In addition, there were a large number of lymphocytes aggregated in this stage.

**Figure 3:**
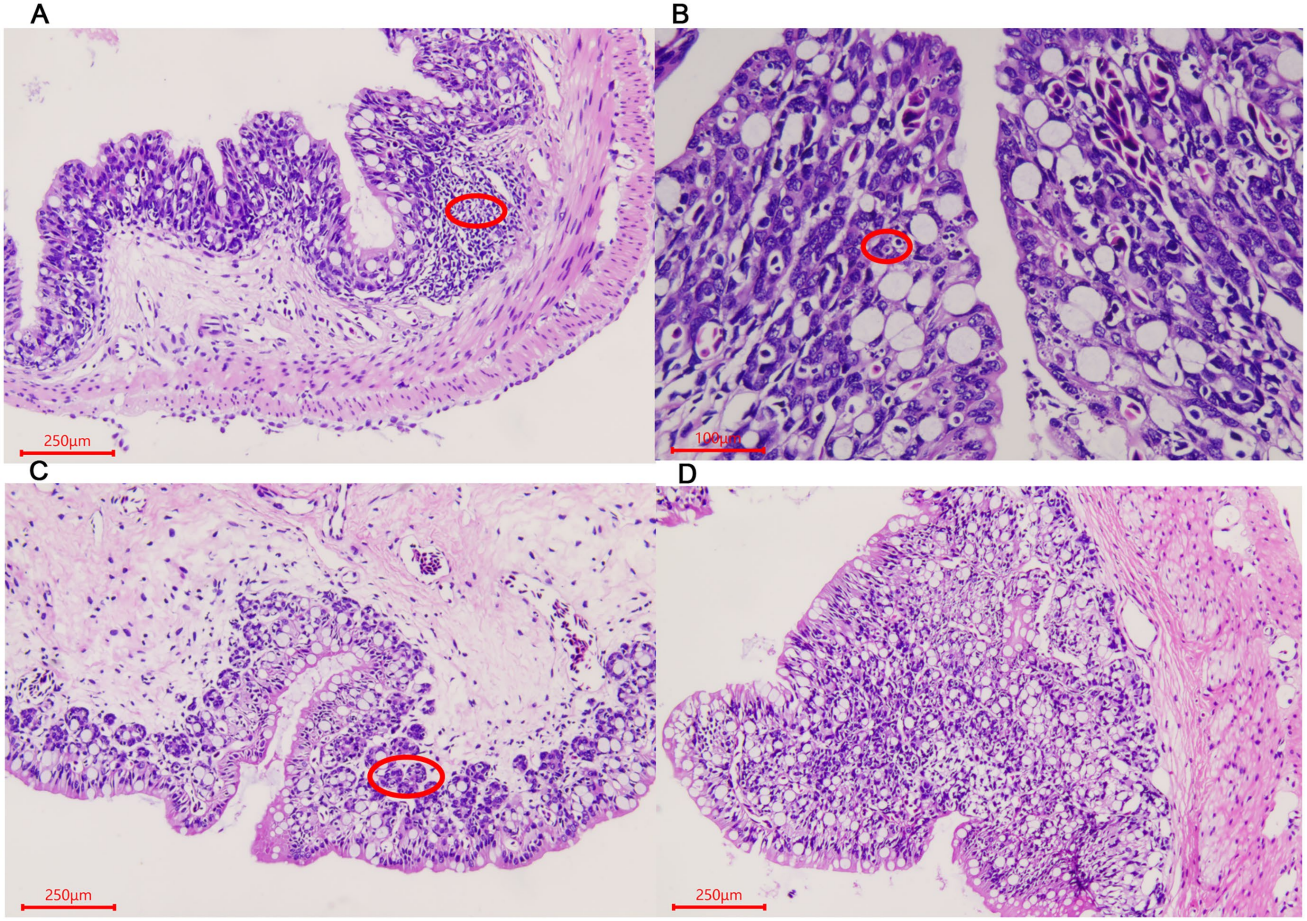
Morphological changes of the rectum in *A. davidianus.* **(A)** Rectum in ADG. **(B)** Rectum in ADY. **(C)** Rectum in ADE. **(D)** Adenoid structure of rectum in ADE. The sections were observed using high power fields of 500-fold magnification with several measurements at different positions in each sample.

For rectum samples, lymph nodes were observed in the submucosa during the ADG period (Figure 3A). Partial epithelial cell necrosis occurred in the villous epithelium in the ADY stage (Figure 3B). The intestinal adenoid structure was observed in the villi during the ADE stage (Figure 3C). The entire intestinal wall thickens during ADS, and the villi become shorter compared to those in other periods (Figure 3D).

### 3.2. Quality assessment and OTU classification of intestinal microbiota

We initially performed a quality screening for high-throughput sequencing data of intestinal microbiota to eliminate erroneous and questionable sequences, which contributed to verifying sequence reliability. A total of 2,824,829 high-quality reads were produced from the data with an average of 62,774 reads per sample (40,308–69,489) and an average length of 252–256 bp (Table 1).

**Table 1:** Statistics of sequence in each sample.

Sample name	Raw PE (#)	Clean tags (#)	Effective tags (#)	AvgLen (nt)	Effective %
ADT.1	101,308	94,073	69,301	255	68.41
ADT.2	100,768	80,775	63,758	254	63.27
ADT.3	107,905	90,226	68,762	254	63.72
ADT.4	80,030	65,983	54,363	256	67.93
ADT.5	76,151	52,868	45,326	256	59.52
ADT.6	60,969	46,926	40,308	254	66.11
ADT.7	82,525	76,883	58,506	254	70.89
ADT.8	100,953	80,845	61,795	255	61.21
ADT.9	111,270	99,848	68,616	254	61.67
ADG.1	98,953	90,992	65,897	253	66.59
ADG.2	106,166	99,808	64,081	253	60.36
ADG.3	101,276	85,942	61,871	253	61.09
ADG.4	99,766	93,940	62,464	253	62.61
ADG.5	99,933	95,332	61,622	253	61.66
ADG.6	100,406	97,713	61,628	253	61.38
ADG.7	104,978	103,333	67,312	253	64.12
ADG.8	60,540	59,720	48,885	253	80.75
ADG.9	99,331	97,963	60,526	253	60.93
ADY.1	101,088	99,812	67,299	253	66.57
ADY.2	106,987	101,732	68,073	253	63.63
ADY.3	98,537	97,225	63,723	253	64.67
ADY.4	106,643	103,450	67,171	253	62.99
ADY.5	108,714	105,960	64,198	253	59.05
ADY.6	99,380	94,419	63,398	253	63.79
ADY.7	102,852	90,903	65,584	253	63.77
ADY.8	97,701	88,630	64,194	253	65.7
ADY.9	80,541	77,103	55,467	253	68.87
ADE.1	95,742	94,082	60,347	252	63.03
ADE.2	100,077	98,453	63,331	252	63.28
ADE.3	101,997	100,245	60,176	252	59
ADE.4	108,372	106,630	69,489	252	64.12
ADE.5	100,517	98,780	61,618	252	61.3
ADE.6	108,086	106,241	64,997	252	60.13
ADE.7	106,566	105,123	68,622	252	64.39
ADE.8	103,684	102,285	65,134	252	62.82
ADE.9	104,706	102,917	62,668	252	59.85
ADS.1	100,827	99,503	60,355	252	59.86
ADS.2	104,600	103,237	65,316	252	62.44
ADS.3	98,551	97,027	62,298	252	63.21
ADS.4	104,411	102,606	67,620	252	64.76
ADS.5	98,451	96,720	63,422	252	64.42
ADS.6	111,531	109,772	65,997	252	59.17
ADS.7	101,881	100,197	67,766	252	66.51
ADS.8	113,859	112,026	69,235	252	60.81
ADS.9	99,731	98,082	62,310	252	62.48

**Figure 4:**
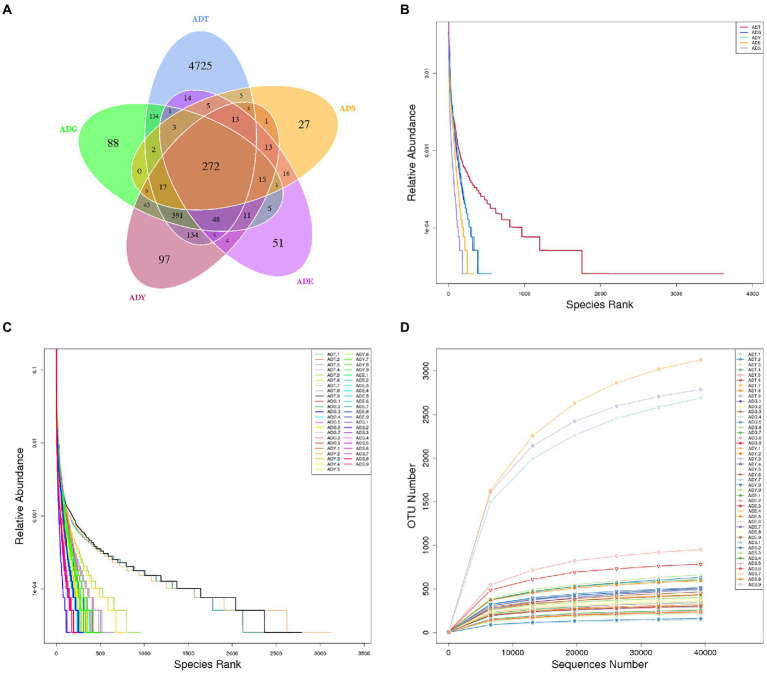
Venn diagrams and feasibility analysis. **(A)** Venn diagrams of the distribution of OTUs in five groups. **(B)** Rank abundance curves for groups. **(C)** Rank abundance curves for samples. **(D)** Rarefaction curves for samples.

The qualified reads were composed of 5,772, 1,033, 1,066, 479, and 393 OTUs in ADT, ADG, ADY, ADE, and ADS based on 97% nucleotide-sequence identity, respectively (Figure 4A). The curves of rarefaction and rank abundance per sample were relatively flat and displayed saturate tendency, which suggested that the depth and evenness of sequences meet the requirements for sequencing and further analysis (Figures 4B–D).

### 3.3. Analysis of microbial community diversity

**Figure 5:**
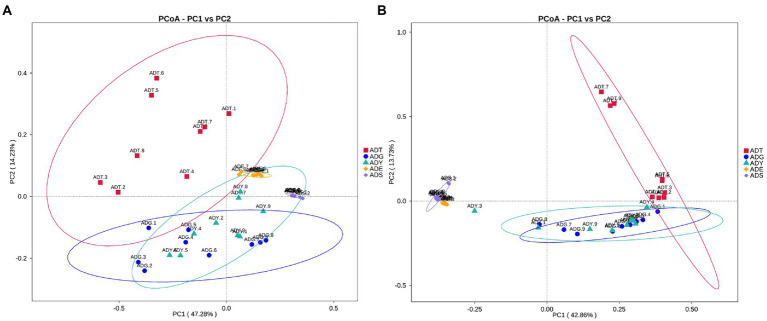
PCoA analysis of the intestinal microbial community in different groups. **(A)** PCoA map on the basis of weighted UniFrac distance. **(B)** PCoA map on the basis of unweighted UniFrac distance.

The alpha diversity of gut microbiota samples showed the goods coverage estimates varied from 99.3% to 99.9% for all samples, which indicated excellent coverage (Table 2). The average Chao1 indices for experimental groups ADT, ADG, ADY, ADE, and ADS were 1594.56, 488.91, 512.81, 354.01, and 262.45, and the corresponding ACE indices were 1589.90, 492.12, 517.26, 350.47, and 264.35, respectively. Moreover, the averages of Shannon indices for these five groups were 7.43, 5.60, 5.55, 4.58, and 3.03, respectively. The Chao1, ACE, Shannon, and Simpson indices for these five groups displayed gradually downward trends, which indicated that the abundance and diversity of the intestinal microbial community reduced as growth. Remarkably, the three diversity indices (ACE, Chao1, and Shannon) of the initial ADT group were much higher than those of other groups. In contrast, significant differences in gut microbiota abundance and diversity were observed between the ADT and other groups. The α-diversity indices revealed a significant difference in the diversity and richness of gut microbiota among different growth groups. Both the weighted and the unweighted principal coordinate analysis (PCoA) plots revealed that the samples in most groups were clustered separately except for some similarity between ADG and ADY, which indicated that the differences existed in gut microbiota for most of the comparative samples (Figure 5).

**Table 2:** Microbial diversity index analysis.

Sample name	Shannon	Simpson	Chao1	ACE	Goods_coverage
ADT	7.43 ± 1.29	0.98 ± 0.02	1594.56 ± 1124.61	1589.90 ± 1120.63	0.993 ± 0.005
ADG	5.60 ± 0.58	0.95 ± 0.02	488.91 ± 105.04	492.12 ± 107.51	0.998 ± 0.001
ADY	5.55 ± 0.26	0.93 ± 0.03	512.81 ± 79.56	517.26 ± 79.88	0.998 ± 0.001
ADE	4.58 ± 0.07	0.86 ± 0.02	354.01 ± 22.48	350.47 ± 14.83	0.999 ± 0.001
ADS	3.03 ± 0.35	0.72 ± 0.04	262.45 ± 42.54	264.35 ± 42.06	0.999 ± 0.001

Shannon, Simpson, Chao1, and ACE were calculated to assess the alpha diversity of the intestinal microbial community. The data are listed as the mean ± SD.

### 3.4. Bacterial community composition in groups

**Figure 6:**
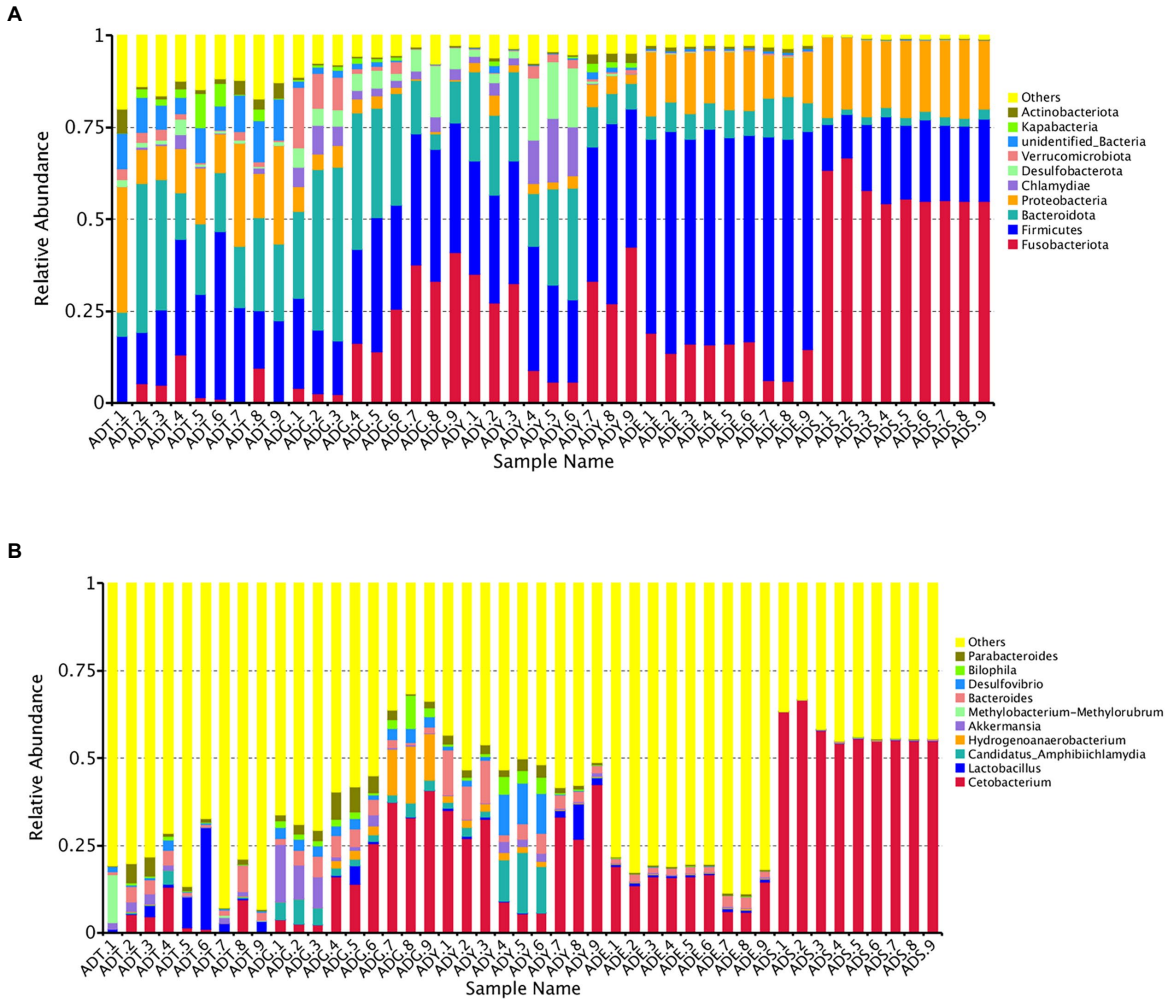
Relative abundance of the gut microbiota of *A. davidianus.* **(A)** Microbial community bar plot of phyla. **(B)** Microbial community bar plot of genera.

Our results showed that the bacterial community comprised 66 phyla, 175 classes, 373 orders, 536 families, 894 genera, and 418 species. The *Fusobacteriota, Firmicutes, Bacteroidota, Proteobacteria, Chlamydiae, Desulfobacterota, Verrucomicrobiota, unidentified_Bacteria, Kapabacteria,* and *Actinobacteriota* were the top 10 phyla for all samples. These phyla constituted the core of the microbiota and accounted for 85.4%–99.3% of the taxonomic groups identified. Especially, the *Firmicutes* represented 59.04% of the totals in ADE, and the *Fusobacteriota* accounted for 57.53% in the ADS group, respectively. The ADT groups were primarily composed of *Firmicutes* (24.53%), *Bacteroidota* (21.52%), and *Proteobacteria* (17.52%). The *Fusobacteriota, Firmicutes, and Bacteroidota* were dominant phyla for the ADG and ADY groups, representing 19.60%, 28.19%, and 26.87% of the totals for the ADG group and 24.18%, 33.21%, and 18.60% for the ADY group. The dominant phyla for ADE and ADS groups were *Fusobacteriota* (13.73% and 57.53%), *Firmicutes* (59.04% and 19.02%), and *Proteobacteria* (14.46% and 20.27%; Figure 6A).

The top 10 genera were *Cetobacterium, Lactobacillus, Candidatus Amphibiichlamydia, Hydrogenoanaerobacterium, Akkermansia, Methylobacterium–Methylorubrum, Bacteroides, Desulfovibrio, Bilophila,* and *Parabacteroides*, which accounted for 17.53%–57.96% of the taxonomic groups identified (Figure 6B). The genus *Cetobacterium* had a significantly higher abundance than other genera, which comprised 3.93%, 19.49%, 24.13%, 13.72%, and 57.52% of the overall bacterial composition in ADT, ADG, ADY, ADE, and ADS groups, respectively. The levels of this genus in the ADS group were significantly higher than in the other groups. *Bacteroides* was the second most abundant at 2.90%, 3.59%, 6.13%, 2.02%, and 0.20% for ADT, ADG, ADY, ADE, and ADS groups, respectively. The other dominant genera were *Hydrogenoanaerobacterium* and *Candidatus Amphibiichlamydia*, which represented 5.57% (ADG) and 5.41% (ADY) of the overall bacterial composition, respectively. In the ADS group, the most numerous genus was *Cetobacterium* at 57.52%, whereas other genera except these top 10 were observed to be predominant for other groups at 51.77%–82.47% of the overall bacterial composition, respectively. The relative abundance of these bacteria was also displayed through a heatmap produced by clustering analysis. The distribution of bacterial genera in each sample could also be observed in the heatmap (Supplementary Figure S1).

### 3.5. Microbial profile and core microbiota of intestinal microflora

Analysis of similarities (ANOSIM) was used to evaluate whether differences between groups (two or more groups) were significantly greater than those within groups (Table 3). The results showed significant and remarkable differences among different groups (*R* > 0, *p* = 0.001), except the ADG–ADY (*p* = 0.127). Although there was no significant difference, the R-values for the ADG–ADY comparison were greater than zero (0.106), indicating potential differences between the ADG and ADY groups.

**Table 3:** ANOSIM of community structure differences between groups.

Group	*R*-value	Value of *p*
ADG-ADY	0.106	0.127
ADS-ADY	0.8059	0.001
ADS-ADG	0.9702	0.001
ADT-ADY	0.4043	0.001
ADT-ADG	0.3707	0.001
ADT-ADS	0.6934	0.001
ADE-ADY	0.8323	0.001
ADE-ADG	0.8803	0.001
ADE-ADS	1	0.001
ADE-ADT	0.6636	0.001

**Figure 7:**
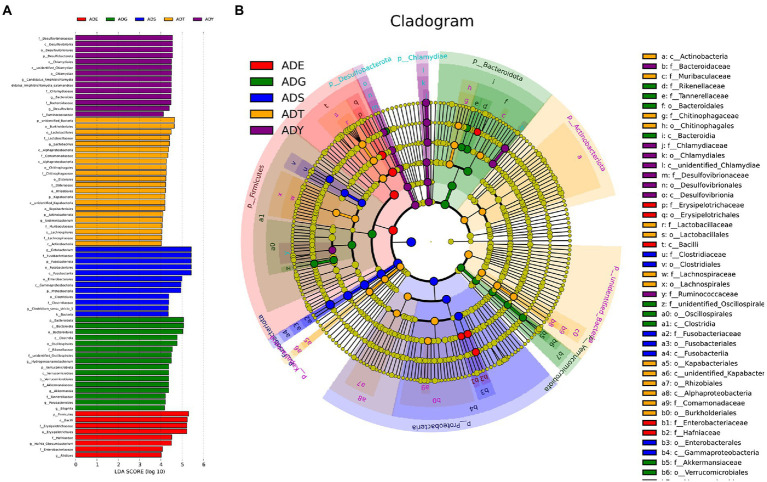
Beta diversity of microbial communities in guts of *A. davidianus* at different growth stages. **(A)** Histogram of linear discriminatory analyses (LDA) score distribution. **(B)** Cladogram of LDA effect size (LEfSe) of microbial taxa (LDA > 4).

To understand the ecosystem of five samples, linear discriminant analysis effect size (LEfSe) analysis was used to uncover the complex system of microbial communities. Meanwhile, linear discriminant analysis was used to identify the differences in all samples (LDA threshold is 4; Figure 7). The results illustrated that there were 43 bacterial clades, consisting of three classes, 15 orders, and 18 families, which were crucial bacterial branches distinguishing giant salamander samples. According to Figure 7A, *Lactobacillus* (4.4) and *Sediminibacterium* (4.1) were significantly enriched in ADT. *Hydrogenoanaerobacterium* (4.5), *Akkermansia* (4.4), *Parabacteroides* (4.2), and *Bilophila* (4.2) were significantly enriched bacterium in ADG. In ADY, three realms showed significant enrichment, which were *Candidatus Amphibiichlamydia* (4.5), *Bacteroides* (4.5), and *Desulfovibrio* (4.4), respectively. *Hafnia Obesumbacterium* (4.5) and *Alistipes* (4.0) were significant in ADE. More bacteria had significant abundance in ADS, such as *Cetobacterium* (5.4) and *Clostridium-sensu-stricto-1* (4.3). In the cladogram, these circles represented different taxonomic levels from phylum to genus, and each small circle represented a classification at that level (Figure 7B). The relative abundance of microbes is proportional to the diameter size of the circle. The significant differences were marked with different colors consistent with corresponding levels except for the yellow which represented no significant difference. These differential microbial groups that play important roles were visually displayed at different levels in the cladogram.

### 3.6. Function prediction

**Figure 8:**
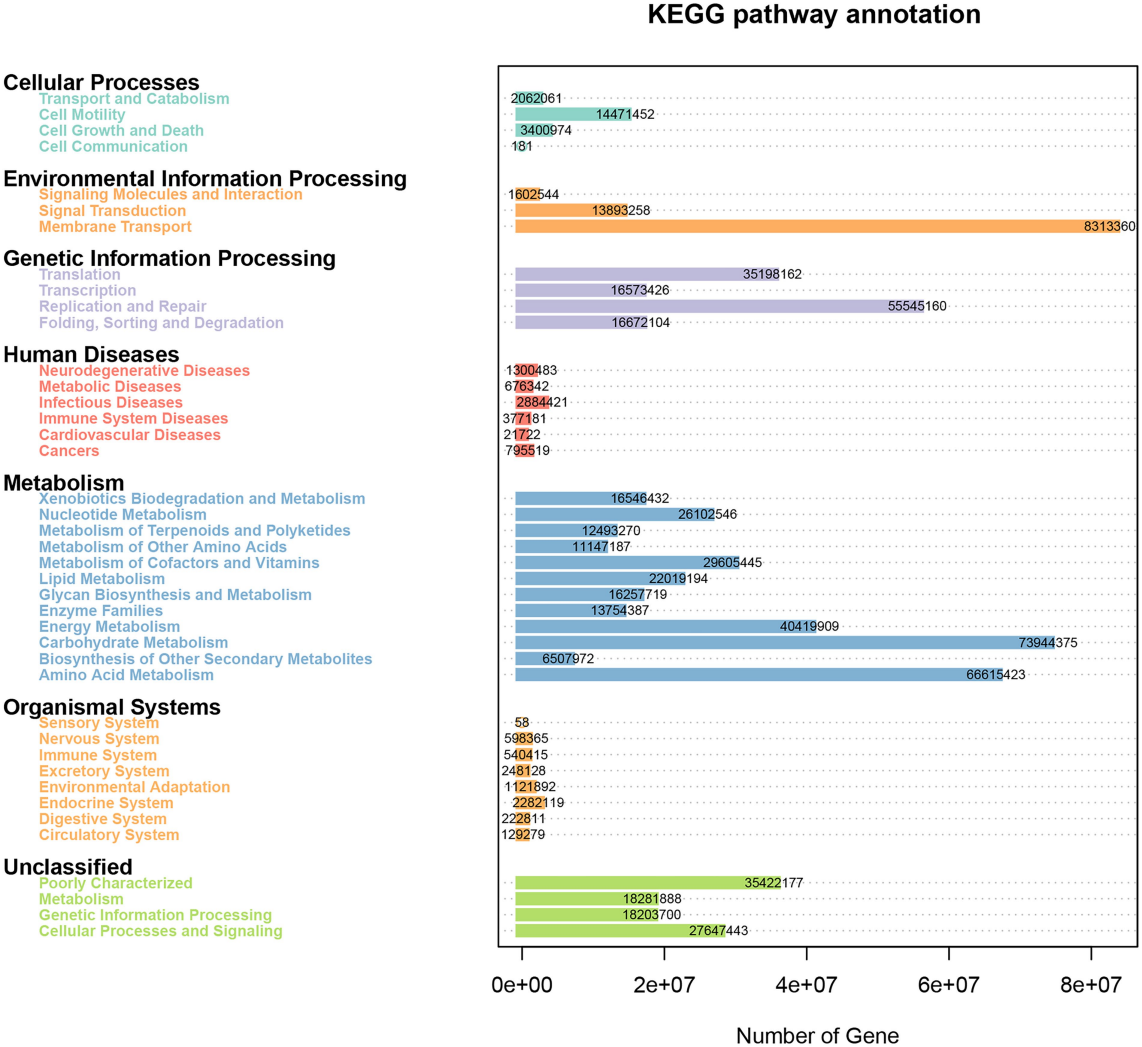
Heatmap of the KEGG pathways annotated by PICRUSt.

The Phylogenetic Investigation of Communities by Reconstruction of Unobserved States (PICRUSt) bioinformatics software package was used to predict the metagenomic function of marker genes based on the KEGG database. As shown in Figure 8, the genes obtained from 16S rRNA sequencing were mainly enriched into 41 KEGG pathways and were classified into seven categories. There were 4, 3, 4, 6, 12, 8, and 4 pathways involved in cellular processes, environmental information processing, genetic information processing, human diseases, metabolism, organismal systems, and unclassified categories, respectively. The membrane transport pathway had the most annotated genes including 8,313,360 genes, which belong to environmental information processing. Moreover, the metabolism category had the most pathways and relatively abundant genes, such as carbohydrate metabolism, amino acid metabolism, and energy metabolism had 73,944,375, 66,615,423, and 40,419,909 genes, respectively. Other enriched pathways, such as replication and repair (55,545,160) and poorly characterized (35,422,177), belonged to genetic information processing and unclassified categories, respectively.

## 4. Discussion

The intestinal tract is the most important digestive and absorption organ of animals. A large number of bacteria were colonized in the intestines and played indispensable roles in maintaining the overall health of the host (Singh et al., 2017). In our histomorphological results, numerous wrinkles in the lining and gradual increases in villi length were observed in *A. davidianus* intestine during growth periods, which contributed to the colonization of microorganisms. The intestinal floras were different among different individuals or the same one in different conditions, which were related to ages, diets, health status, and ranging profile (Korpela et al., 2021; Liu et al., 2021; Spencer et al., 2021; Sztandarski et al., 2022). High-throughput sequencing was used to investigate the gut and lung prokaryotic community profiles of adult Chinese giant salamanders at age 3 (Wu et al., 2019). In addition, the previous gut microbiota report of *A. davidianu*s provided much microbial information related to age changes from age 1 to 4 (Zhang et al., 2018). However, studies on earlier ages, histomorphological observation, developmental effects, and disease-related pathogen identification are still lacking. These intestinal microbes are comprised of both beneficial and harmful members. The balance maintenance of intestinal flora and an increase in probiotics proportion could effectively keep the host healthy (Aziz et al., 2013). Therefore, the acquisition of composition and abundance of intestinal flora is well helpful to disease prevention and treatment (Feng et al., 2018).

Gut microbiota plays an important role in the growth and development of host animals with huge abundance and complex structures. We found that the alpha diversity indices, such as OTU number, Shannon, Simpson, Chao1, and ACE, were decreased as *A. davidianus* grew, which indicated the species diversity and abundance of intestinal flora reduced. These values in the ADT stage were significantly higher than in other periods, which may be deduced that the number of beneficial bacteria increased while conditional pathogens were reduced. The phenomena of lymphoid follicles accumulation and adenoid structure that existed in submucosa first observed in ADG also contributed to the elimination of harmful microbes in guts. A previous study also revealed that phylogeny plays the most important role in the formation of microbial communities, rather than food and environment (Bai et al., 2021). Previous studies verified that aging is a multifactorial process and would influence many principal physiological systems, including the gastrointestinal system (Mabbott et al., 2015). In addition, histomorphological changes in the gut at different ages or stages were found in mice (Yang et al., 2019). In humans, both the composition and stability of gut microbiota were reported to change with age (Li et al., 2016). However, the relationship between gut microbiota and gut histomorphological needs further studies.

The proportion of *Fusobacteriota* in the ADS group was significantly higher than in other groups, and there was a gradually increased tendency during growth periods except for ADE. More detailed analysis showed that *Cetobacterium* was the dominant genus in this phylum. On the contrary, the levels of *Bacteroidota, unidentified_Bacteria,* and *Kapabacteria* displayed a gradual decline. *Fusobacteriota, Firmicutes*, and *Actinobacteriota* are the dominant intestinal phyla for all animals. These similarities suggested that these microorganisms are important participants of host functions, such as normal digestion, absorption, and immune responses. *Cetobacterium*, an anaerobic bacteria belonging to the core microbiota of fish gut, was also the most dominant genus in *A. davidianus*. *Lactobacillus*, known as beneficial bacteria, was common in the gastrointestinal tract of most aquatic animals (Liu et al., 2021). They could convert large amounts of hexose substrates into pyruvate and then generate the final lactate *via* the glycolytic pathway (Dempsey and Corr, 2022). More importantly, the abundance of *Candidatus Amphibiichlamydia* genus in early growth periods (ADT, ADG, and ADY) was much higher than in later phases (ADE and ADS). We speculated that it may be the result of *A. davidianus* development, as these salamanders develop, their small intestine increases in complexity, and the wrinkles and partmentalization may play key roles in the abundance changes of *Candidatus Amphibiichlamydia*. This species has attracted much attention as a special pathogen for amphibian diseases (Martel et al., 2013), which may be a promising potential marker for the prediction of *A. davidianus* diseases. Although none of the *A. davidianus* tadpoles showed signs of clinical disease, more experiments related to pathogenic conditions need to be performed considering a high prevalence of 71% in bullfrogs (*Lithobates catesbeianus*).

In beta diversity, the result of PCoA showed that there was a very similar species composition between ADG and ADY, which indicated a closer relationship in the two samples compared to others. This result was also verified by statistical analysis of ANOSIM and LEfSe, further indicating the differences of intergroup were greater than intragroup. These results were consistent with previous high-throughput sequencing results in zebrafish gut microbiota (Xiao et al., 2021). Host development overwhelmed environmental effects in governing fish gut microbial community succession from larvae to adult fish stages due to host genetics, immunology, and gut nutrient niches. This study is another example of reduced abundance and diversity of the intestinal microbial community as growth.

Maintaining a healthy gut is key to disease prevention in animals. Metabolic activities of microorganisms would generate many important nutrients, such as short-chain fatty acids, vitamins, and amino acids, which would affect host health. The succinate and secondary bile acids produced by *Parabacteroides distasonis* played key roles in the modulation of host metabolism, and disturbance of intestinal flora was closely related to the occurrence of obesity, diabetes, and hyperlipidemia (Wang et al., 2019). The enriched KEGG pathways of annotated genes were also oriented to multiple functions of intestinal microorganisms in the giant salamander. Different growth and developed stages could have differential intestinal structures, which may influence intestine flora. The intestinal flora, in turn, also could affect the intestinal development and immune function of *A. davidianus*. Keeping the balance of intestinal flora would effectively help the host maintain health status.

## 5. Conclusion

This study investigated the changes in the intestinal microbial community in *A. davidianus* from larvae to adult stages. The results revealed that the diversity and abundance of gut microbiota had significant alterations that were characterized by declining levels of growth. Most of the top 10 phyla and genera had significant differences among different groups, except for a similar microbial community in ADG–ADY. These genes annotated in intestinal microbes were mainly enriched into KEGG pathways including cellular processes and environmental information processing, which played important roles in growth metabolism, nutrient absorption, and immune regulation. In addition, *Candidatus Amphibiichlamydia*, a special species for amphibians, was a promising potential indicator of gut microbiota stability. These findings expanded our current understanding of the succession of gut microbiota across *A. davidianus* growth and also provided new insights into the breeding monitoring of other aquatic animals.

## Data availability statement

The data presented in the study are deposited in the NCBI repository, accession number PRJNA903076. The data is publicly available with accession number of PRJNA903076 and website: https://www.ncbi.nlm.nih.gov/bioproject/PRJNA903076.

## Ethics statement

The animal study was reviewed and approved by the Ethics Committee of Chongqing University of Arts and Sciences.

## Author contributions

MC and HD carried out biochemical assays, performed 16S rRNA amplicon analysis, and drafted and revised the manuscript. HS, MH, and WF participated in the design of the study, analyzed the data, and revised the manuscript. XL, WS, and JH conceived the study and revised the manuscript. All authors contributed to the article and approved the submitted version.

## Funding

This study was supported by the Science and Technology Research Project of Chongqing Municipal Education Commission (grant no. KJQN201801301), the Foundation and Advanced Research Project of Chongqing Science and Technology Commission (grant nos. cstc2020jscx-msxmX0055 and cstc2019jscx-gksbX0147), and Chongqing Talents Programme (grant no. CQYC20200309221).

## Conflict of interest

The authors declare that the research was conducted in the absence of any commercial or financial relationships that could be construed as a potential conflict of interest.

## Publisher’s note

All claims expressed in this article are solely those of the authors and do not necessarily represent those of their affiliated organizations, or those of the publisher, the editors and the reviewers. Any product that may be evaluated in this article, or claim that may be made by its manufacturer, is not guaranteed or endorsed by the publisher.

## References

[ref1] Aziz Q. Doré J. Emmanuel A. Guarner F. Quigley E. M. (2013). Gut microbiota and gastrointestinal health: current concepts and future directions. Neurogastroenterol. Motil. 25, 4–15. doi: 10.1111/nmo.12046, PMID: 23279728

[ref2] Bai S. Zhang P. Zhang C. Du J. Du X. Zhu C.. (2021). Comparative study of the gut microbiota among four different marine mammals in an aquarium. Front. Microbiol. 12:769012. doi: 10.3389/fmicb.2021.769012, PMID: 34745077 PMC8567075

[ref3] Benson A. K. Kelly S. A. Legge R. Ma F. Low S. J. Kim J.. (2010). Individuality in gut microbiota composition is a complex polygenic trait shaped by multiple environmental and host genetic factors. Proc. Natl. Acad. Sci. U. S. A. 107, 18933–18938. doi: 10.1073/pnas.1007028107, PMID: 20937875 PMC2973891

[ref4] Bokulich N. A. Subramanian S. Faith J. J. Gevers D. Gordon J. I. Knight R.. (2013). Quality-filtering vastly improves diversity estimates from Illumina amplicon sequencing. Nat. Methods 10, 57–59. doi: 10.1038/nmeth.2276, PMID: 23202435 PMC3531572

[ref5] Caporaso J. G. Kuczynski J. Stombaugh J. Bittinger K. Bushman F. D. Costello E. K.. (2010). QIIME allows analysis of high-throughput community sequencing data. Nat. Methods 7, 335–336. doi: 10.1038/nmeth.f.303, PMID: 20383131 PMC3156573

[ref6] Dempsey E. Corr S. C. (2022). Lactobacillus spp. for gastrointestinal health: current and future perspectives. Front. Immunol. 13:840245. doi: 10.3389/fimmu.2022.840245, PMID: 35464397 PMC9019120

[ref7] Feng W. Ao H. Peng C. (2018). Gut microbiota, short-chain fatty acids, and herbal medicines. Front. Pharmacol. 9:1354. doi: 10.3389/fphar.2018.01354, PMID: 30532706 PMC6265305

[ref8] Gerritsen J. Smidt H. Rijkers G. T. de Vos W. M. (2011). Intestinal microbiota in human health and disease: the impact of probiotics. Genes Nutr. 6, 209–240. doi: 10.1007/s12263-011-0229-7, PMID: 21617937 PMC3145058

[ref9] Gui L. Chinchar V. G. Zhang Q. (2018). Molecular basis of pathogenesis of emerging viruses infecting aquatic animals. Aquacult. Fisheries 3, 1–5. doi: 10.1016/j.aaf.2017.12.003

[ref10] He G. Z. (2012). Entamoeba histolytica: cloning, expression and evaluation of the efficacy of a recombinant amebiasis cysteine proteinase gene (ACP1) antigen in minipig. Exp. Parasitol. 130, 126–129. doi: 10.1016/j.exppara.2011.11.007, PMID: 22154977

[ref11] Jiang N. Fan Y. Zhou Y. Liu W. Ma J. Meng Y.. (2015). Characterization of Chinese giant salamander iridovirus tissue tropism and inflammatory response after infection. Dis. Aquat. Org. 114, 229–237. doi: 10.3354/dao02868, PMID: 26036830

[ref12] Korpela K. Kallio S. Salonen A. Hero M. Kukkonen A. K. Miettinen P. J.. (2021). Gut microbiota develop towards an adult profile in a sex-specific manner during puberty. Sci. Rep. 11:23297. doi: 10.1038/s41598-021-02375-z, PMID: 34857814 PMC8640005

[ref13] Langille M. G. I. Zaneveld J. Caporaso J. G. McDonald D. Knights D. Reyes J. A.. (2013). Predictive functional profiling of microbial communities using 16S rRNA marker gene sequences. Nat. Biotechnol. 31, 814–821. doi: 10.1038/nbt.2676, PMID: 23975157 PMC3819121

[ref14] Ley R. E. Hamady M. Lozupone C. Turnbaugh P. J. Ramey R. R. Bircher J. S.. (2008). Evolution of mammals and their gut microbes. Science 320, 1647–1651. doi: 10.1126/science.1155725, PMID: 18497261 PMC2649005

[ref15] Li H. Qi Y. Jasper H. (2016). Preventing age-related decline of gut compartmentalization limits microbiota Dysbiosis and extends lifespan. Cell Host Microbe 19, 240–253. doi: 10.1016/j.chom.2016.01.008, PMID: 26867182 PMC5106289

[ref16] Liang G. (2007). Chinese Giant salamander captive breeding models in Shaanxi Province and primary assessment. J. Econ. Anim. 11, 234–237. doi: 10.13326/j.jea.2007.04.015

[ref17] Liu C. Zhao L. P. Shen Y. Q. (2021). A systematic review of advances in intestinal microflora of fish. Fish Physiol. Biochem. 47, 2041–2053. doi: 10.1007/s10695-021-01027-3, PMID: 34750711

[ref18] Mabbott N. A. Kobayashi A. Sehgal A. Bradford B. M. Pattison M. Donaldson D. S. (2015). Aging and the mucosal immune system in the intestine. Biogerontology 16, 133–145. doi: 10.1007/s10522-014-9498-z 24705962

[ref19] Magoč T. Salzberg S. L. (2011). FLASH: fast length adjustment of short reads to improve genome assemblies. Bioinformatics 27, 2957–2963. doi: 10.1093/bioinformatics/btr507, PMID: 21903629 PMC3198573

[ref20] Martel A. Adriaensen C. Sharifian-Fard M. Vandewoestyne M. Deforce D. Favoreel H.. (2013). The novel 'Candidatus Amphibiichlamydia ranarum' is highly prevalent in invasive exotic bullfrogs (*Lithobates catesbeianus*). Environ. Microbiol. Rep. 5, 105–108. doi: 10.1111/j.1758-2229.2012.00359.x, PMID: 23757138

[ref21] Meng Y. Ma J. Jiang N. Zeng L. B. Xiao H. B. (2014). Pathological and microbiological findings from mortality of the Chinese giant salamander (*Andrias davidianus*). Arch. Virol. 159, 1403–1412. doi: 10.1007/s00705-013-1962-6, PMID: 24385158

[ref22] Quast C. Pruesse E. Yilmaz P. Gerken J. Schweer T. Yarza P.. (2013). The SILVA ribosomal RNA gene database project: improved data processing and web-based tools. Nucleic Acids Res. 41, D590–D596. doi: 10.1093/nar/gks1219, PMID: 23193283 PMC3531112

[ref23] Schmitt V. H. Schmitt C. Hollemann D. Weinheimer O. Mamilos A. Kirkpatrick C. J.. (2019). Tissue expansion of lung bronchi due to tissue processing for histology – a comparative analysis of paraffin versus frozen sections in a pig model. Pathol. Res. Pract. 215:152396. doi: 10.1016/j.prp.2019.03.024, PMID: 30954348

[ref24] Siezen R. J. Kleerebezem M. (2011). The human gut microbiome: are we our enterotypes? Microb. Biotechnol. 4, 550–553. doi: 10.1111/j.1751-7915.2011.00290.x, PMID: 21848611 PMC3819005

[ref25] Singh R. K. Chang H. W. Yan D. Lee K. M. Ucmak D. Wong K.. (2017). Influence of diet on the gut microbiome and implications for human health. J. Transl. Med. 15:73. doi: 10.1186/s12967-017-1175-y, PMID: 28388917 PMC5385025

[ref26] Song M. Zeng J. Jia T. Gao H. Zhang R. Jiang J.. (2019). Effects of sialylated lactulose on the mouse intestinal microbiome using Illumina high-throughput sequencing. Appl. Microbiol. Biot. 103, 9067–9076. doi: 10.1007/s00253-019-10169-7, PMID: 31659420

[ref27] Spencer C. N. McQuade J. L. Gopalakrishnan V. McCulloch J. A. Vetizou M. Cogdill A. P.. (2021). Dietary fiber and probiotics influence the gut microbiome and melanoma immunotherapy response. Science 374, 1632–1640. doi: 10.1126/science.aaz7015, PMID: 34941392 PMC8970537

[ref28] Sztandarski P. Marchewka J. Konieczka P. Zdanowska-Sąsiadek Ż. Damaziak K. Riber A. B.. (2022). Gut microbiota activity in chickens from two genetic lines and with outdoor-preferring, moderate-preferring, and indoor-preferring ranging profiles. Poultry Sci. 101:102039. doi: 10.1016/j.psj.2022.102039, PMID: 35952604 PMC9385685

[ref29] Tilg H. Kaser A. (2011). Gut microbiome, obesity, and metabolic dysfunction. J. Clin. Invest. 121, 2126–2132. doi: 10.1172/jci58109, PMID: 21633181 PMC3104783

[ref30] Turnbaugh P. J. Quince C. Faith J. J. McHardy A. C. Yatsunenko T. Niazi F.. (2010). Organismal, genetic, and transcriptional variation in the deeply sequenced gut microbiomes of identical twins. Proc. Natl. Acad. Sci. U. S. A. 107, 7503–7508. doi: 10.1073/pnas.1002355107, PMID: 20363958 PMC2867707

[ref31] Turnbaugh P. J. Ridaura V. K. Faith J. J. Rey F. E. Knight R. Gordon J. I. (2009). The effect of diet on the human gut microbiome: a metagenomic analysis in humanized gnotobiotic mice. Sci. Transl. Med. 1:6ra14. doi: 10.1126/scitranslmed.3000322, PMID: 20368178 PMC2894525

[ref32] Turvey S. T. Chen S. Tapley B. Liang Z. Wei G. Yang J.. (2021). From dirty to delicacy? Changing exploitation in China threatens the world's largest amphibians. People Nat. 3, 446–456. doi: 10.1002/pan3.10185

[ref33] Wang K. Liao M. Zhou N. Bao L. Ma K. Zheng Z.. (2019). Parabacteroides distasonis alleviates obesity and metabolic dysfunctions via production of succinate and secondary bile acids. Cell Rep. 26, 222–235.e5. doi: 10.1016/j.celrep.2018.12.028, PMID: 30605678

[ref34] Wilson K. (2001). Preparation of genomic DNA from bacteria. Curr. Protoc. Mol. Biol. 56, 2.4.1–2.4.5. doi: 10.1002/0471142727.mb0204s56 18265184

[ref35] Wu Z. Gatesoupe F.-J. Zhang Q. Wang X. Feng Y. Wang S.. (2019). High-throughput sequencing reveals the gut and lung prokaryotic community profiles of the Chinese giant salamander (Andrias davidianus). Mol. Biol. Rep. 46, 5143–5154. doi: 10.1007/s11033-019-04972-8, PMID: 31364018

[ref36] Xiao F. Zhu W. Yu Y. He Z. Wu B. Wang C.. (2021). Host development overwhelms environmental dispersal in governing the ecological succession of zebrafish gut microbiota. NPJ Biofilms Microbi. 7:5. doi: 10.1038/s41522-020-00176-2, PMID: 33469034 PMC7815754

[ref37] Yang M. Liu Y. Xie H. Wen Z. Zhang Y. Wu C.. (2019). Gut microbiota composition and structure of the Ob/Ob and Db/Db mice. Int. J. Endocrinol. 2019, 1394097–1394099. doi: 10.1155/2019/1394097, PMID: 30984260 PMC6432735

[ref38] Zhang P. Chen Y. Q. Liu Y. F. Zhou H. Qu L. H. (2003). The complete mitochondrial genome of the Chinese giant salamander, Andrias davidianus (Amphibia: Caudata). Gene 311, 93–98. doi: 10.1016/s0378-1119(03)00560-2, PMID: 12853142

[ref39] Zhang M. Gaughan S. Chang Q. Chen H. Lu G. Wang X.. (2018). Age-related changes in the gut microbiota of the Chinese giant salamander (Andrias davidianus). Microbiology 8:e778. doi: 10.1002/mbo3.778, PMID: 30585426 PMC6612560

[ref40] Zhu L. Zhu W. Zhao T. Chen H. Zhao C. Xu L.. (2021). Environmental temperatures affect the gastrointestinal microbes of the Chinese Giant salamander. Front. Microbiol. 12:543767. doi: 10.3389/fmicb.2021.543767, PMID: 33815302 PMC8017128

