# Supplementary material for: Changes of intestinal microbiota in the giant salamander (Andrias davidianus) during growth based on high-throughput sequencing
Source: Front Microbiol. 2023 Mar 16;14:1052824. doi: 10.3389/fmicb.2023.1052824 (PMC10061097; doi:10.3389/fmicb.2023.1052824)
Supplement: Supplementary file 1 [file Image_1.pdf]

Supplementary Material

1 Supplementary Figures

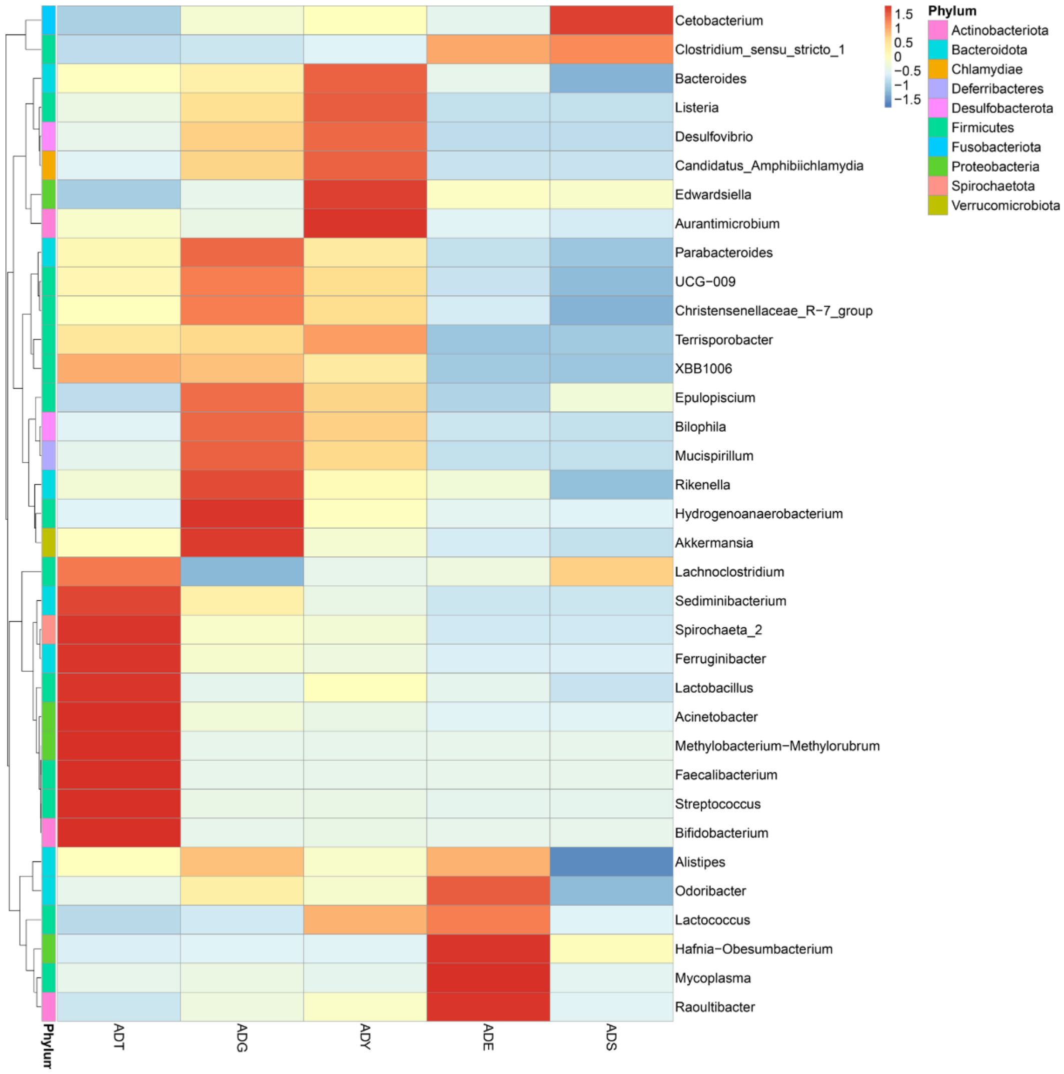

Supplementary Figure 1. Heatmap of the relative richness of bacterial communities at the genus level. Each color-block in the heatmap represents the relative richness of a bacterial genus in a sample.
